# Pattern recognition in SARS cases: insights from *t*-SNE and k-means clustering applied to COVID-19 symptomatology

**DOI:** 10.3389/frai.2025.1536486

**Published:** 2025-03-27

**Authors:** Julliana Gonçalves Marques, Bruno Motta de Carvalho, Luiz Affonso Guedes, Márjory Da Costa-Abreu

**Affiliations:** ^1^Department of Informatics and Applied Mathematics, Federal University of Rio Grande do Norte, Natal, Brazil; ^2^Department of Computer Engineering and Automation, Federal University of Rio Grande do Norte, Natal, Brazil; ^3^School of Computing and Digital Technologies, Sheffield Hallam University, Sheffield, United Kingdom

**Keywords:** COVID-19, *t*-SNE, k-means, symptoms, pattern recognition

## Abstract

**Introduction:**

Despite the end of the SARS-CoV-2 pandemic, the medical field continues to address several lasting effects, the most notable being long COVID. However, COVID-19 presents another specific challenge that complicates diagnosis: the similarity of its symptoms with those of other viral diseases, particularly among various SARS strains. This overlap makes it difficult to identify distinct and meaningful symptom patterns as they develop. This study proposes a dimensionality reduction approach combined with a clustering technique to visually analyse structural similarities among SARS-infected individuals, aiming to determine whether aspects such as case progression and diagnosis impact these patterns.

**Methods:**

This analysis utilised the t-Distributed Stochastic Neighbour Embedding (*t*-SNE) algorithm for dimensionality reduction, combined with Gower's distance to handle categorical data, and k-means clustering. The study focused on symptoms, case progression, and diagnoses of SARS-CoV-2 and unspecified SARS cases using data from the Brazilian SARS dataset for São Paulo State during 2020 and 2021. The process began with a visual analysis aimed at identifying structural patterns in the symptom data, highlighting potential similarities between COVID-19 patients and those diagnosed with unspecified SARS. Following this, an intra-cluster analysis was performed to investigate the common features that defined each cluster, providing insights into shared characteristics among grouped individuals.

**Results:**

The analysis revealed that both diagnoses share substantial similarities, particularly in the presence or absence of COVID-19-related symptoms, even when the majority of individuals were diagnosed with unspecified SARS.

**Discussion:**

The analysis is crucial, as Brazil was one of the countries most severely affected by the pandemic, experiencing profound impacts across multiple dimensions.

## 1 Introduction

The COVID-19 pandemic, beginning in December 2019, had a lasting impact with multiple waves, setting it apart from earlier pandemics. Health systems were often overwhelmed as cases surged beyond diagnostic capacities. Research has advanced our understanding of the virus and its variants, revealing how symptom patterns key to public recognition of the disease–differ across strains. These symptoms became crucial for timely diagnosis, especially during critical phases of the pandemic. COVID-19 shares symptoms with other respiratory viruses, complicating diagnosis. This highlights the need for techniques that uncover intrinsic patterns in data, with Machine Learning (ML) approaches playing a key role in disease recognition.

In the medical field, large and complex datasets require dimensionality reduction methods to extract relevant information. Additionally, exploring data structure is crucial, often using clustering techniques like *t*-SNE and k-means. *t*-SNE preserves local structure while mapping high-dimensional data to lower dimensions, aiding in pattern visualisation. Meanwhile, k-means groups similar data points, helping identify clusters of patients with common symptoms.

Several studies have employed *t*-SNE to identify symptom patterns in COVID-19 data. Shi et al. (2021) applied it to classify asymptomatic, presymptomatic, and symptomatic cases among 2,980 hospitalised patients in Wuhan between February and April 2020. Their results revealed 13 cell clusters among 17 patients and showed that *t*-SNE effectively plotted CD+8 T cells, linking their exhaustion to COVID-19 progression. Another study Eskandarian et al. (2023) analysed clinical features associated with mortality in 3,008 COVID-19 patients hospitalised in Iran between March and November 2020. Using *t*-SNE and other dimensionality reduction techniques, the study investigated symptoms such as fever, myalgia, dizziness, seizure, and abdominal pain, ultimately finding no significant correlation between mortality and gender or symptoms. Ta et al. (2023) aimed to identify clinical subgroups among 11,313 hospitalised COVID-19 patients between March 2020 and December 2021. The study identified 20 patient subgroups of varying severity, with *t*-SNE effectively visualising distinct clusters. One notable subgroup (SG8) exhibited higher rates of dyspnea (56.4%), cough (37.3%), and asthenia (23.3%), but lower rates of sepsis (5.5%).

K-means has been applied to cluster COVID-19 symptoms and related data. Utomo (2021) compared K-means and K-medoids for clustering COVID-19 spread in Indonesia using data from 175,095 confirmed cases. K-means outperformed K-medoids based on the Davies-Bouldin Index, forming more cohesive clusters. Chimbunde et al. (2023) used K-means to identify risk factor clusters in 392 COVID-19 patients in South Africa. Four main groups emerged, with Clusters 3 and 4 showing high case fatality rates (62.8% and 75.6%), while Cluster 2 had the lowest (44%). Wahyuddin and Pradana (2021) applied K-means to cluster COVID-19 symptoms in Indonesia using 14 key symptoms from August 2021. Three clusters were identified: one with mild symptoms (e.g., fever and cough), another with moderate to severe cases, and a third with gastrointestinal symptoms, which were less frequent.

Most studies on COVID-19 focus on diagnostics, often analysing genetic material or additional patient data, while fewer have examined symptom patterns, particularly in comparison to other SARS strains. Understanding these patterns is crucial for distinguishing COVID-19 from similar infections, especially in ambiguous cases. In previous work Marques et al. (2024), we analysed symptom patterns in SARS-CoV-2 and unspecified SARS cases using the Apriori algorithm to identify potential undetected COVID-19 cases. Here, we propose using *t*-SNE with Gower's distance for categorical data and k-means clustering to explore structural similarities among SARS-infected individuals. In addition to symptoms, we examine case progression and diagnosis. Experiments were conducted using SARS data from São Paulo State's Brazilian SARS database (2020–2021), covering periods of both high and low infection rates.

## 2 Materials and methods

### 2.1 Data acquisition

For the experiments, COVID-19 and unspecified SARS cases from 2020 (1,007,052 individuals) and 2021 (976,633 individuals) were obtained from the SARS database available at SUS (2020b), a publicly accessible database related to the Brazilian health situation. These periods were chosen because the first 2 years of the pandemic registered the highest number of infections and deaths worldwide, especially in Brazil. In addition to symptom variables, the datasets contained social demographics, risk factors, comorbidities, and laboratory findings for all states. Most of the feature values are categorical, including symptom features, which are represented by “y” (yes) and ‘n' (no) to indicate the presence or absence of the symptom, respectively. A description of all database variables can be found in SUS (2020a). However, in it is important to point the inability to distinguish COVID-19-negative samples from other types. It is due to limited access to testing, as testing was available to individuals with moderate and severe cases. For this reason, we decided to compare SARS-CoV-2 cases with the unspecified SARS category, which is proportionally significant in both periods.

**Figure 1:**
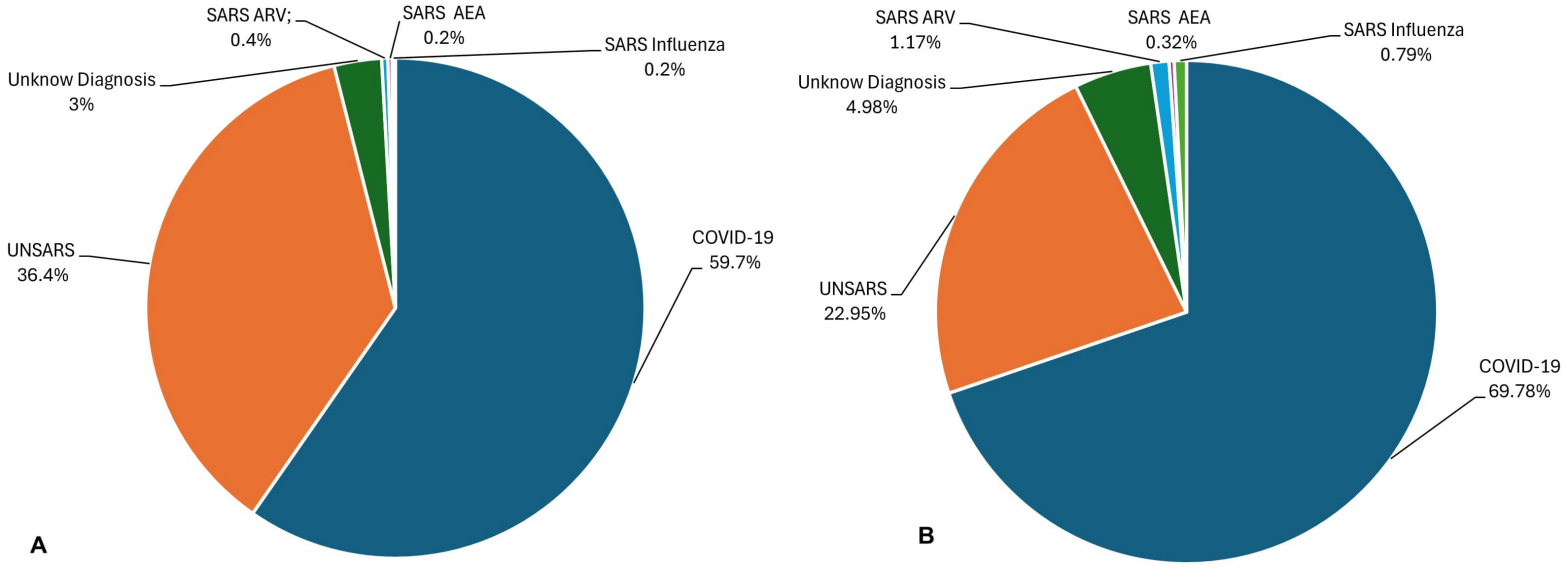
**(A)** Final Case Classification (diagnostic) of all SARS database in 2020. **(B)** Final Case Classification (diagnostic) of all SARS database in 2021.

In Figure 1 it is show all final case classification in 2020 (Figure 1A) and 2021 (Figure 1B). Brazilian health system classify the cases into five categories: SARS caused by influenza, SARS caused by another respiratory virus (SARS ARV), SARS caused by another aetiological agent (SARS AEA), SARS caused by COVID-19, and unspecified SARS that refers to cases in which no alternative aetiological agent is identified, making it impossible to collect or process clinical samples for laboratory diagnosis or confirm through clinical–epidemiological criteria, clinical imaging, or clinical diagnosis.

### 2.2 Feature selection

Thus, given Brazil's vast geographic size, COVID-19 exhibited varying behaviours across different regions of the country. Therefore, it was decided to use data from the state of São Paulo. Due to its large population and influx of tourists, São Paulo is believed to represent the most dynamic COVID-19 scenario in the country, making its data a valuable source of information for the experiments. The peak periods of infection in 2020 and 2021 years were analysed. In 2020, the highest number of cases was recorded in July (25,975 cases) and October (10,157) had the lowest number of cases, while in 2021, the highest number of cases occurred in March (72,824) and the lowest number of cases happened in November (2,754) (Lorenz et al., 2021). The 12 symptoms used in the experiments are described in Marques et al. (2024).

### 2.3 Categorical data

Categorical data refers to variables that represent distinct categories or groups, often non-numeric. Examples include gender and answer *yes* or *no*, for example. These variables are typically classified into nominal (unordered categories, like colours) or ordinal (ordered categories, like rankings) types. Categorical data is found in various fields, such as social sciences, public health, where responses or features are naturally grouped into categories rather than continuous numerical values (Agresti, 2012).

The information labelled into distinct classes is useful for tasks like demographic analysis or classification but lacks inherent numerical meaning, necessitating specific handling techniques in machine learning, where algorithms typically expect numerical inputs (Hancock and Khoshgoftaar, 2020). Common approaches to represent categorical data include direct usage or transformation into numerical form through methods such as one-hot encoding, label encoding, and embedding techniques (Cerda and Varoquaux, 2020; Rodríguez et al., 2018; Shah et al., 2022; Yang, 2005). For unsupervised learning techniques, such as dimensionality reduction and clustering, distance measures are essential to evaluate similarity or dissimilarity between categorical values (Alamuri et al., 2014; Santos and Zárate, 2015; Ben Ali and Massmoudi, 2013; D'Orazio, 2021). In the experiments, Gower's distance was chosen (Gower, 1971) due to its ability to handle mixed data types (categorical and numerical) effectively, ensuring an accurate representation of the complex feature space in the dataset.

### 2.4 Gower's distance

The Gower distance is a versatile dissimilarity measure designed to handle mixed data types, including numerical, categorical, and even ordinal variables. Proposed by J.C. Gower in 1971 (Gower, 1971), it calculates the distance between data points by normalising individual attribute differences. For categorical attributes, it assigns a value of 0 for a match and 1 for a mismatch, while for continuous data, it scales differences relative to their range. This makes Gower's distance particularly useful in clustering and dimensionality reduction techniques when working with heterogeneous datasets. Its flexibility allows it to accommodate diverse types of variables within a single unified measure.

Thus, *x*_*ik*_ represents the value of attribute *k* for instance *i*. The similarity between two instances is compared for each attribute *k* assigning an index *s*_*ijk*_, where *s*_*ijk*_ = 0 if the two instances are completely different, or a positive real number if they share some level of similarity. δ_*ijk*_ = 1 if instances *i* and *j* can be compared for attribute *k* (*x*_*ik*_, *x*_*jk*_) and 0 otherwise. *w*_*k*_ represents the weight for attribute *k*. The similarity can be calculated as follows (Ben Ali and Massmoudi, 2013):


(1) $$\begin{array}{l} S_{ij}=\overset{N}{\underset{k=1}{\sum }}s_{ijk}\delta_{ijk}w_{k}/\overset{N}{\underset{k=1}{\sum }}\delta_{ijk}w_{k} \end{array}$$

For binary and categorical attributes, *s*_*ijk*_ = 0 if *x*_*ik*_ = *x*_*jk*_, and *s*_*ijk*_ = 1, otherwise, while for continuous attributes, $s_{ijk}=\frac{\left|x_{ik}-x_{jk}\right|}{max_{l}x_{lk}-min_{l}x_{lk}}$ where *l* runs for all non-missing values for the attribute *k*.

### 2.5 *t*-distributed stochastic neighbour embedding

The *t*-Distributed Stochastic Neighbour Embedding (*t*-SNE) is a non-linear, unsupervised, manifold-based Feature Extraction (FE) technique that maps high-dimensional data into a lower-dimensional space (2 or 3 dimensions), while preserving the important structures of the original dataset. Its formulation is given as follows Balamurali and Melkumyan (2016):

Given a set of of *N* high-dimensional objects *X*_1_, ..., *X*_*n*_, *t*-SNE first computes probabilities *p*_*ij*_ that are proportional to the similarity between objects *X*_*i*_ and *X*_*j*_, as follows:


(2) $$\begin{array}{l} p_{j|i}=\frac{exp\left(-||x_{i}-x_{j}|{|}^{2}/2{\sigma_{i}}^{2}\right)}{\underset{k\neq i}{\sum }exp\left(-||x_{i}-x_{k}|{|}^{2}/2{\sigma_{i}}^{2}\right)}, \end{array}$$

(3) $$\begin{array}{l} p_{i,j}=\frac{p_{j|i}+p_{i|j}}{2N}. \end{array}$$

The bandwidth of the Gaussian kernels, denoted as σ, is adjusted so that the perplexity of the conditional distribution matches a specified perplexity through a binary search. Consequently, the bandwidth is adapted based on the data density: smaller σ_*i*_ values are applied in regions where the data is denser.

The objective of the *t*-SNE is to learn a *d*-dimensional map *Y*_1_, ..., *Y*_*n*_ (with $Y_{i}\in {\mathbb{R}}^{d}$) that reflects the similarities *p*_*ij*_. To achieve this, it calculates the similarities *q*_*ij*_ between two points on the map, *y*_*i*_ and *y*_*j*_, using a similar method. Specifically, *q*_*ij*_ is defined as:


(4) $$\begin{array}{l} q_{i,j}=\frac{{\left(1+||y_{i}-y_{j}|{|}^{2}\right)}^{-1}}{\underset{k\neq i}{\sum }{\left(1+||y_{k}-y_{j}|{|}^{2}\right)}^{-1}} \end{array}$$

Here, a heavy-tailed Student *t*-distribution is used to measure similarities between low-dimensional points, allowing dissimilar objects to be positioned far apart on the map. The locations of the points *y*_*i*_ in the map are determined by minimising the (non-symmetric) Kullback-Leibler divergence between the distribution *Q* and the distribution *P*, which is expressed as:


(5) $$\begin{array}{l} KL=\left(P||Q\right)=\underset{i\neq j}{\sum }p_{i,j}\log\frac{p_{i,j}}{q_{i,j}} \end{array}$$

The minimisation of the Kullback-Leibler (KL) divergence with respect to the points *y*_*i*_ is carried out using gradient descent. This optimisation process produces a map that accurately captures the similarities between the high-dimensional inputs.

The *t*-SNE algorithm was implemented in Python, using Scikitlearn (2007) library. The parameters were kept at their default settings, except for the perplexity parameter, which was adjusted based on the divergence. A range of divergence values was defined, and *t*-SNE was executed across this range. The final perplexity = 100 was chosen based on a curve plotting the divergence values, identifying the point where divergence stabilises.

Following the visualisation provided by *t*-SNE, an intra-cluster analysis is performed. The number of clusters visually identified in the *t*-SNE plot is used to determine the parameter *k* for the *k*-means algorithm, which is then applied using the embedding generated by *t*-SNE.

### 2.6 k-means

*k*-means clustering is a widely used unsupervised machine learning technique that partitions a dataset into *k* clusters, where each data point is assigned to the cluster with the nearest centroid. This method is particularly popular in both scientific and industrial applications, such as gene expression clustering and text classification. Its simplicity and effectiveness have contributed to its broad usage in various fields. Following a description a brief description of its functioning (Dubey and Choubey, 2017; Jain, 2010):

Given a dataset *X* = {*x*_1_, *x*_2_, ..., *x*_*n*_}, where each data point *x*_*i*_ is a vector in ℝ^*d*^, the goal of *k*-means is to partition these *n* points into *k* clusters *C*_1_, *C*_2_, ..., *C*_*K*_. The algorithm aims to minimise the following *objective function*, known as the Sum of Squared Errors (SSE) or Within-cluster Sum of Squares (WCSS) (Bishop and Nasrabadi, 2006).


(6) $$\begin{array}{l} J=\overset{K}{\underset{k=1}{\sum }}\underset{x_{i}\in C_{k}}{\sum }||x_{i}-\mu_{k}|{|}^{2} \end{array}$$

where: μ_*k*_ is the centroid (mean) of cluster *C*_*k*_, *x*_*i*_ represents a data point in cluster *C*_*k*_, $||x_{i}-\mu_{k}|{|}^{2}$ is the squared Euclidean distance between *x*_*i*_ and μ_*k*_.

The *k*-means algorithm consists of the following steps:

1. Initialisation: Choose *k* initial centroids (either randomly or using methods like k-means++ to improve convergence).
2. Assignment: Assign each data point to the nearest centroid based on the Euclidean distance.
3. Update: Recalculate the centroids of each cluster by computing the mean of all points assigned to it.
4. Convergence: Repeat the assignment and update steps until the centroids no longer change or the algorithm converges to a predefined tolerance.

The *k*-means algorithm was implemented in Python, using Scikitlearn (2007) library. The parameters were kept at their default settings, except for the *k* parameter, that was adjusted based on the number of cluster identified visually through *t*-SNE visualisation.

## 3 Results

In this section, the results obtained using the *t*-SNE and k-means algorithms are presented. Periods of both high and low infection peaks were selected from the first two years of the pandemic. In 2020, the selected periods were July and October, while in 2021, we selected March and November. Initially, a preliminary analysis of individuals visual arrangement will be conducted to explore the most evident similarities between confirmed COVID-19 diagnoses and unspecified diagnoses. Subsequently, an intra-cluster analysis will be performed, focusing on aspects such as the most prevalent diagnoses, patient outcomes (recovery, death, or death from other causes), and the most common symptoms.

Although definitive conclusions about the shapes of the structures cannot be drawn, the formation of distinct agglomerations resembling clusters is evident. Therefore, for educational purposes, we will refer to these agglomerations as clusters, as their arrangement aligns with the concept.

**Figure 2:**
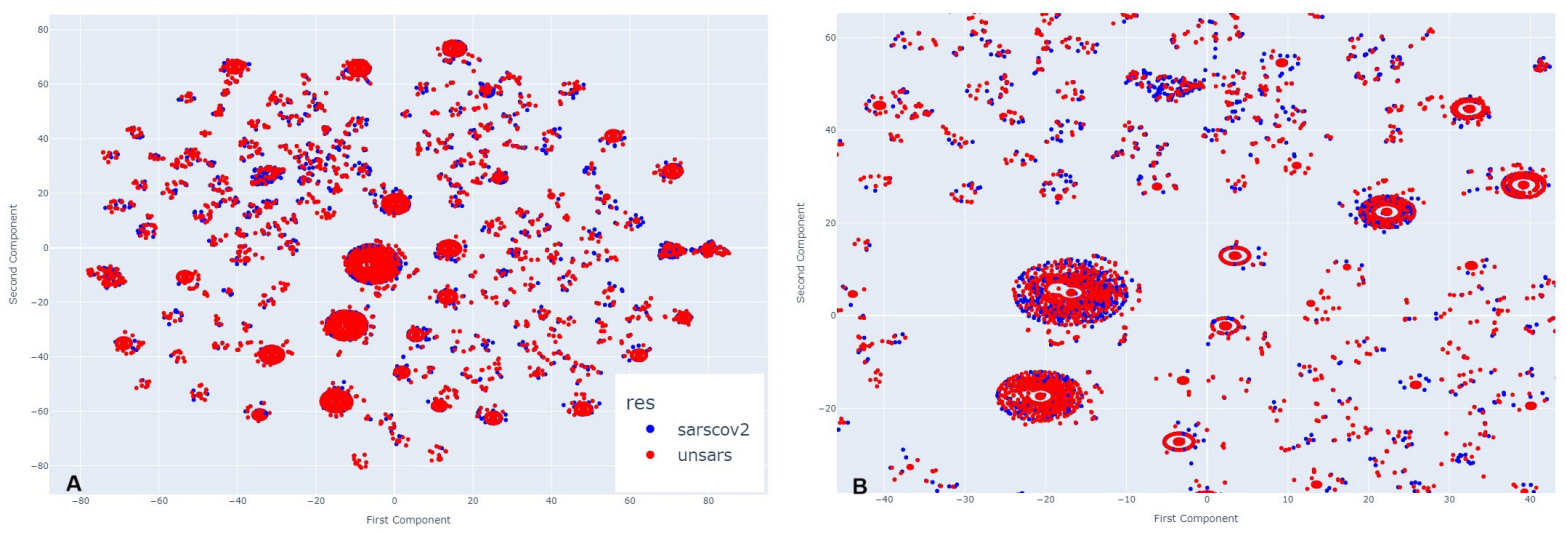
**(A)** Clusters formed by COVID-19 and Unspecified SARS symptoms in July 2020. **(B)** The clusters formed by COVID-19 and Unspecified SARS symptoms in July 2020 after applying a zoom to the figure.

Figure 2A illustrates the clusters formed from COVID-19 and unspecified SARS symptoms in July 2020. As *t*-SNE preserves the proximity relationships and distances between similar data points, it does not allow for direct interpretation of clusters as in traditional clustering analysis. However, the closer the data points, the more similar they are. A closer inspection of the clusters in Figure 2B reveals significant overlap between both diagnoses at the centre and surrounding areas of the clusters. The two diagnoses are so similar that it is nearly impossible to visually distinguish them. In some instances, unspecified SARS individuals are more noticeable, while in others, COVID-19 individuals stand out, but overall, both diagnoses overlap within the clusters and other agglomerations on the plot.

**Figure 3:**
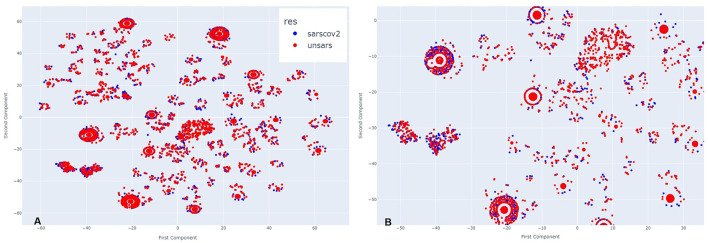
**(A)** Clusters formed by COVID-19 and Unspecified SARS symptoms in October 2020. **(B)** The clusters formed by COVID-19 and Unspecified SARS symptoms in October 2020 after applying a zoom to the figure.

In November, a period of lower peak infection cases in 2020, Figure 3A shows that the clusters are arranged differently, with more poorly formed agglomerations compared to the previous period, suggesting that they appear more dispersed at first glance. However, upon zooming in (Figure 3B), the same pattern observed earlier re-emerges. COVID-19 and unspecified SARS individuals are so closely positioned that, depending on the cluster, it is nearly impossible to distinguish the overlapping points. This indicates that even during a period of fewer reported cases, the symptomatology of these diagnoses remains highly similar.

**Figure 4:**
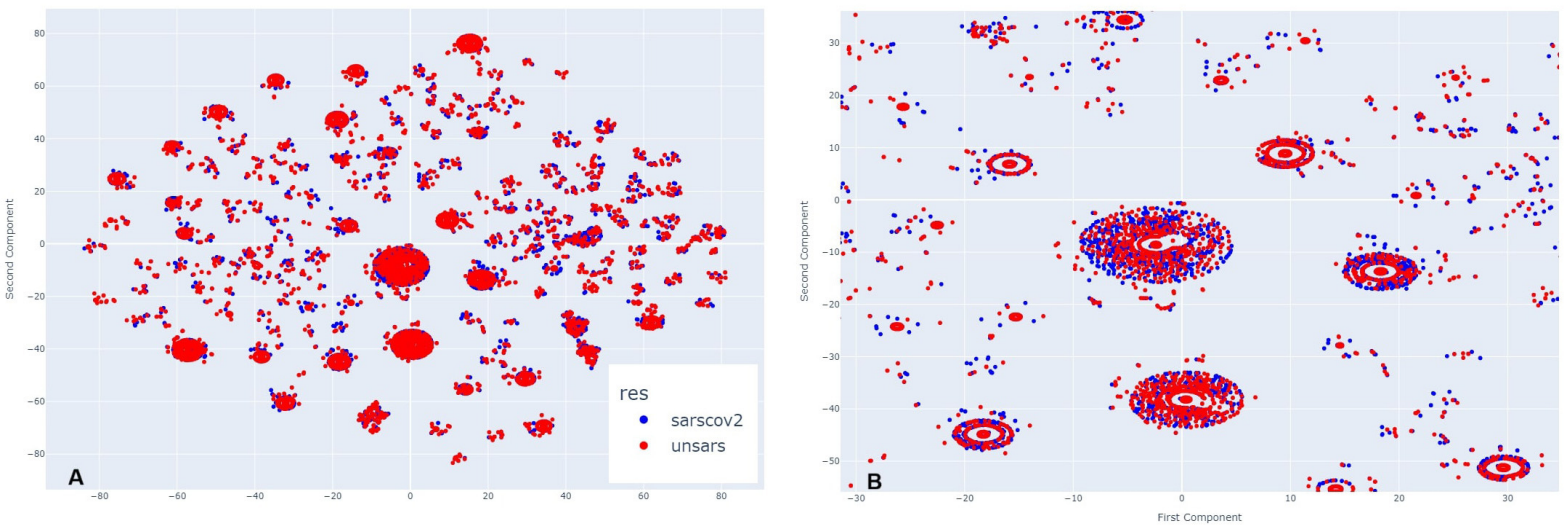
**(A)** Clusters formed by COVID-19 and Unspecified SARS symptoms in March 2021. **(B)** The clusters formed by COVID-19 and Unspecified SARS symptoms in March 2021 after applying a zoom to the figure.

Figure 4 illustrates March 2021. Analysing side Figure 4A, it is evident that more clusters have formed compared to the previous year, along with visible overlapping points. Upon zooming in Figure 4B, the same pattern appears, but with a greater number of COVID-19 individuals compared to those diagnosed with unspecified SARS. This visual impression aligns with the period of higher case reporting between the first two years of the pandemic. Despite less overlap between points, even during this period of more reported SARS-CoV-2 cases than other SARS strains, the individuals still exhibit very similar symptomatology.

**Figure 5:**
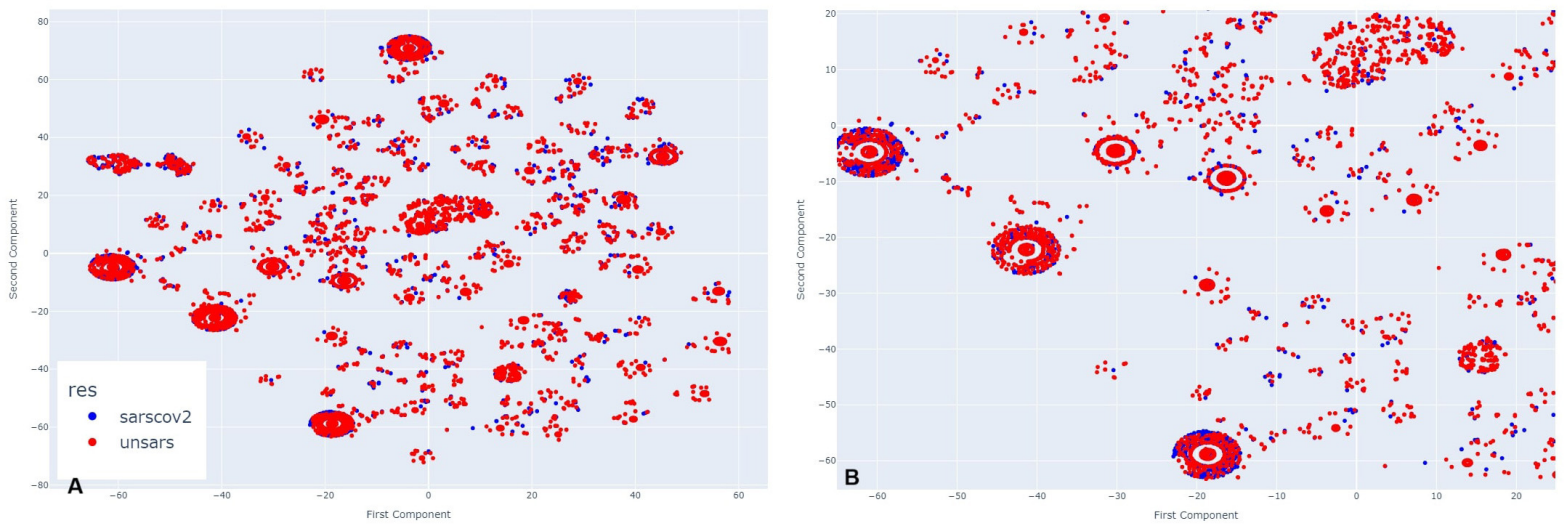
**(A)** Clusters formed by COVID-19 and Unspecified SARS symptoms in November 2021. **(B)** The clusters formed by COVID-19 and Unspecified SARS symptoms in November 2021 after applying a zoom to the figure.

In Figure 5A, which represents the period of a low peak of cases in 2021, a similar visual pattern to March is observed, but, in contrast to previous periods, there appear to be more unspecified SARS individuals than COVID-19 cases. Upon zooming in (Figure 5B), this observation is confirmed in the majority of agglomerations. During this period, the introduction of a new SARS-CoV-2 variant, alongside vaccination efforts, may have contributed to a decrease in SARS-CoV-2-like cases. However, even in this scenario, it remains clear across all clusters that there is significant overlap between the diagnoses.

By examining all periods, it becomes evident that the symptomatology of both SARS-CoV-2 and unspecified SARS is strikingly similar. Regardless of the presence or absence of specific symptoms, individuals from both groups share similar characteristics. To uncover these shared features, an intra-cluster analysis was conducted, focusing on significant cluster sizes by examining both the largest and smallest clusters.

### 3.1 Intra-cluster analysis

**Figure 6:**
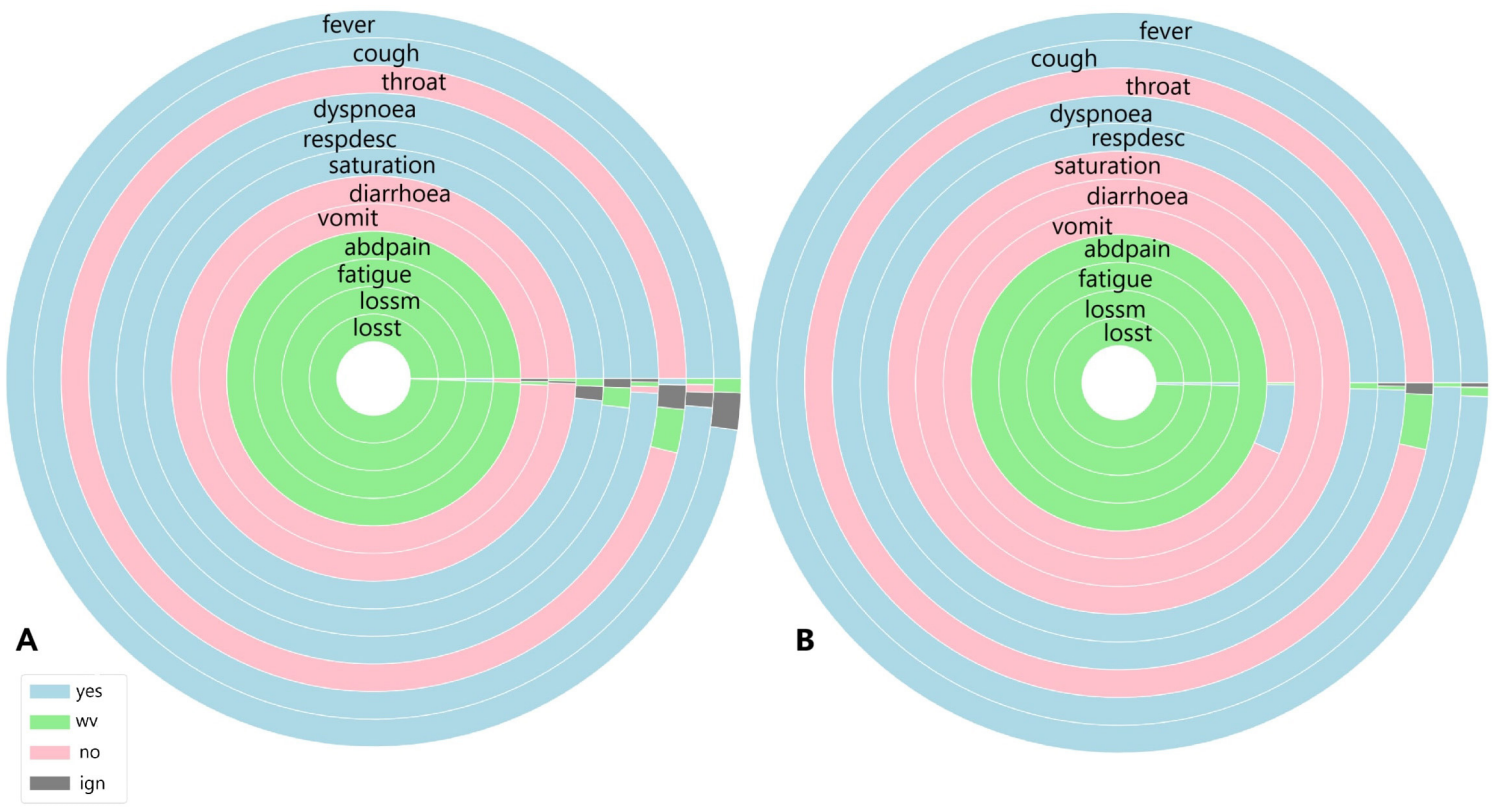
**(A)** Symptoms exhibited by individuals in the cluster with the highest and **(B)** the lowest COVID-19 and unspecified SARS infections reported in July 2020.

**Figure 7:**
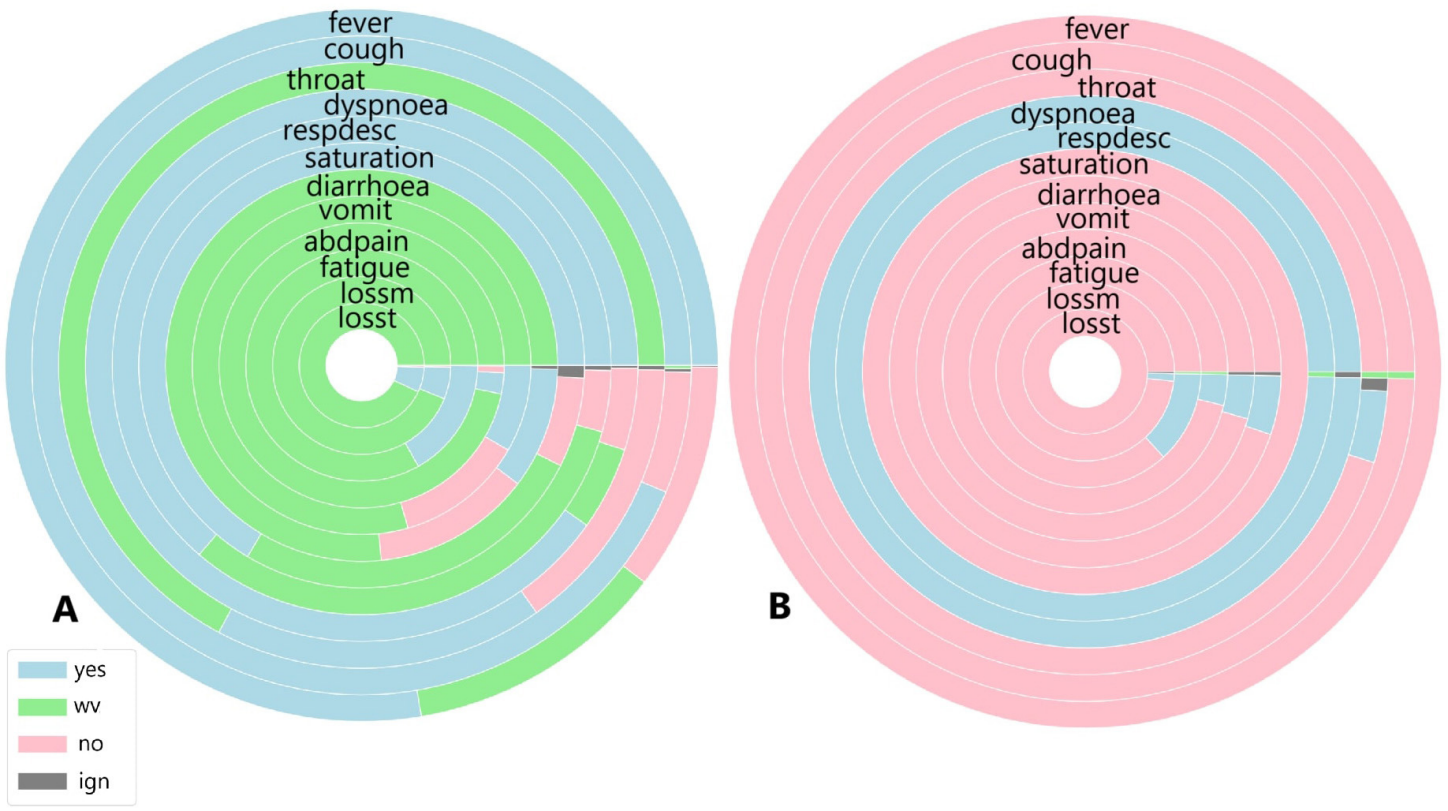
**(A)** Symptoms exhibited by individuals in the cluster with the highest and **(B)** the lowest COVID-19 and unspecified SARS infections reported in October 2020.

**Figure 8:**
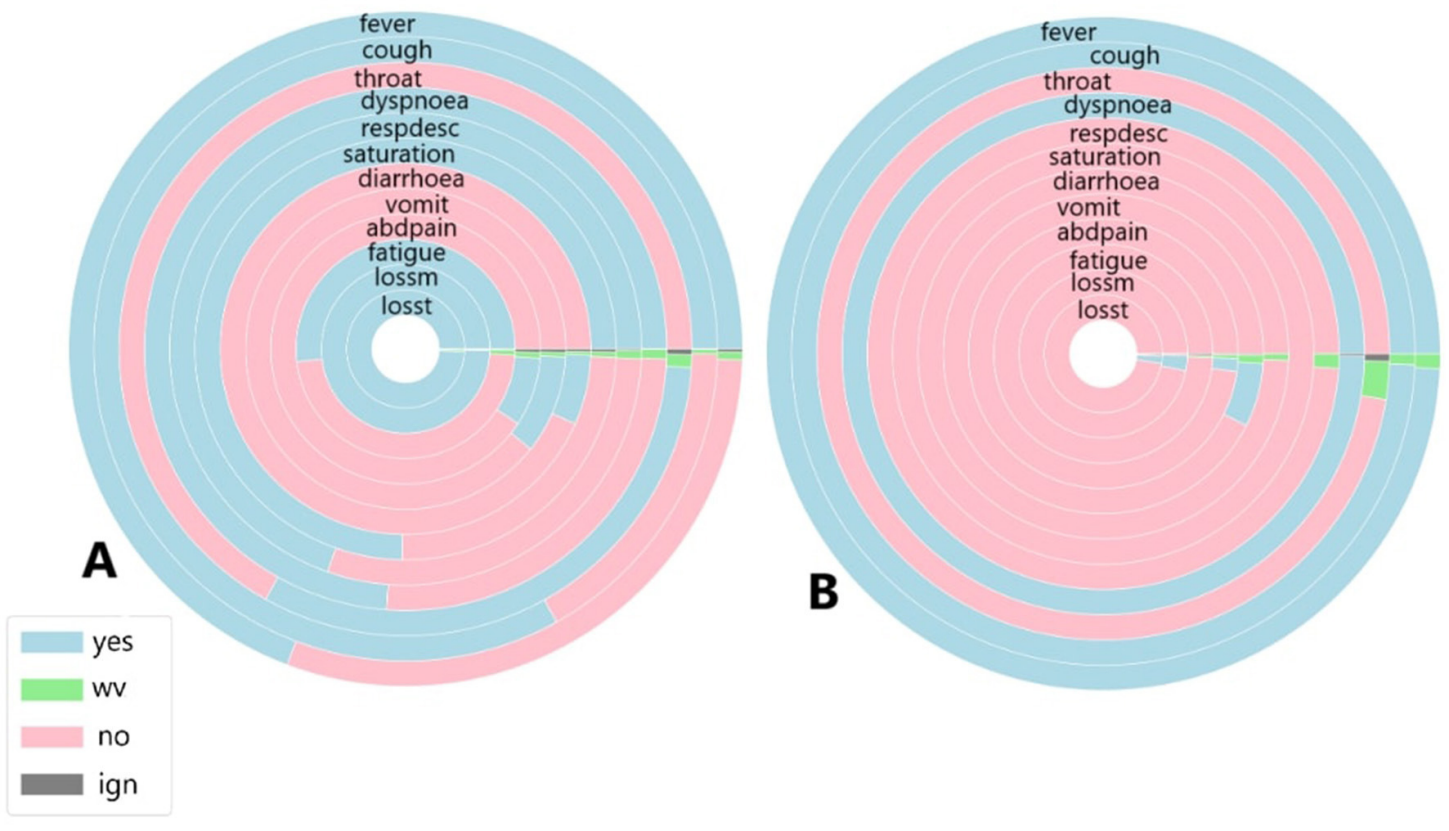
**(A)** Symptoms exhibited by individuals in the cluster with the highest and **(B)** the lowest COVID-19 and unspecified SARS infections reported in March 2021.

**Figure 9:**
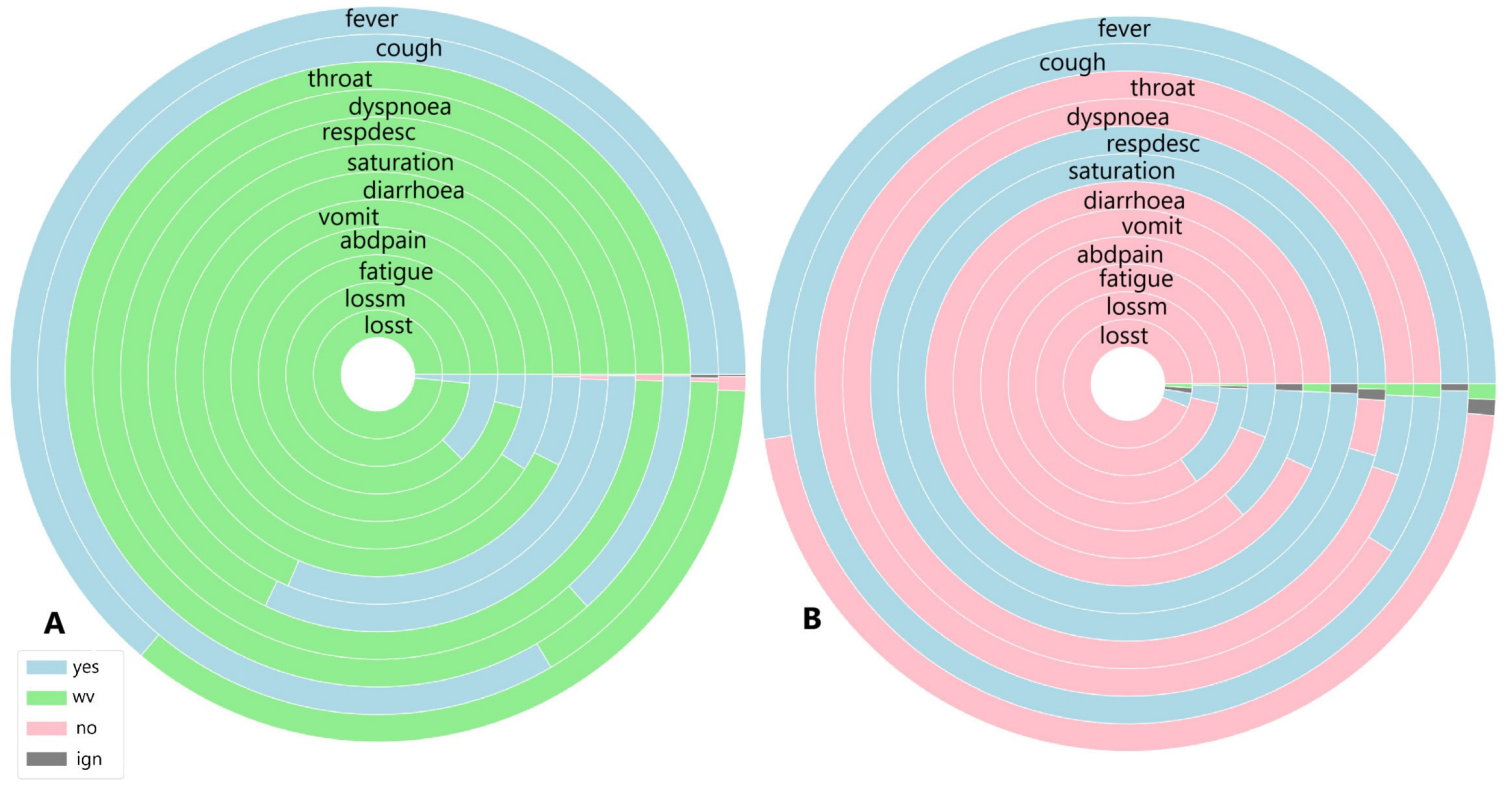
**(A)** Symptoms exhibited by individuals in the cluster with the highest and **(B)** the lowest COVID-19 and unspecified SARS infections reported in November 2021.

To identify similarities between COVID-19 and unspecified SARS patients, the analysis considers not only symptoms but also diagnoses and case outcomes. Figures 6, 7, 8, 9 illustrate the prevalence of each symptom using Donut Charts. In these charts, the blue segment represents the presence of a symptom (“yes”), the pink segment indicates its absence “no”), the green segment denotes symptoms with no recorded value (“wv”), and the grey segment corresponds to symptoms ignored (“ign”) either by the patient or healthcare professionals. Each symptom line is colour-coded to visually reflect the proportion of these categories. The data presented in the charts remains unaltered. Specifically, side A of each figure represents the largest cluster for months with a high number of SARS cases, indicating the group with the highest number of individuals. In contrast, side B corresponds to the smallest cluster for months with fewer SARS cases, reflecting the group with the lowest number of individuals.

Figure 6 shows the symptoms presented by individuals in both the largest cluster (Figure 6A) and the smallest cluster (Figure 6B) in July 2020. Analysing the largest cluster (Figure 6A), the majority of individuals from both diagnostic groups displayed symptoms such as fever, cough, dyspnea, respiratory discomfort, and blood oxygen saturation issues, along with other flu-like and respiratory symptoms typically associated with mild to moderate COVID-19 cases. However, symptoms like sore throat and gastrointestinal issues (such as vomiting and abdominal pain) were either absent or unreported by the patients. Additionally, there was no recorded information on loss of smell and taste, symptoms strongly associated with SARS-CoV-2 infection. In the smaller cluster (Figure 6B), a similar pattern was observed. Most individuals exhibited fever and cough, along with other respiratory symptoms, although blood oxygen saturation was not affected in this group. Likewise, gastrointestinal symptoms, fatigue, and loss of smell and taste were either absent or not documented by the patients. Although symptoms are not the only factor in diagnosis, they are often the most visible indicators for both healthcare professionals and the general population. Additionally, as previously noted (Figure 1), there was no significant circulation of other viruses causing SARS during the periods analysed.

Given this context, focusing solely on symptomatology and examining each cluster individually, both clusters display similar percentages of COVID-19-related deaths, with 11% in Cluster A and 14% in Cluster B. Recovery rates were also comparable, at 80.5% in Cluster A and 81% in Cluster B. However, a marked difference emerged in the proportion of patients with unspecified SARS cases: in the larger Cluster A, these cases represented 17% of patients, while in the smaller Cluster B, they accounted for nearly half, at 41%. This suggests that, despite differing diagnoses, nearly half of the patients in Cluster B exhibited similar symptoms, death rates, and recovery outcomes to those in Cluster A.

In October, 29 clusters were identified. Cluster 1 contained the highest number of individuals, totalling 1,210, while Cluster 6 had the fewest, with 289 individuals. In the largest cluster, 676 individuals were diagnosed with COVID-19, and 534 had an unspecified SARS diagnosis. Among these, 833 individuals recovered, 225 died from COVID-19, and for 7, the cause of death was unrecorded. In the smallest cluster, 106 individuals were diagnosed with COVID-19, and 183 had an unspecified SARS diagnosis. Of these, 199 recovered, 50 died from COVID-19, and 35 died from other causes.

Figure 7 illustrates the symptoms reported by individuals in both the largest cluster (Figure 7A) and the smallest cluster (Figure 7B) in October 2020. Upon analysing the largest cluster (Figure 7A), a different pattern emerges. For the most frequently reported symptoms fever, cough, dyspnoea, respiratory discomfort, and oxygen saturation a substantial portion of individuals did not present these symptoms, particularly in cases of fever and respiratory symptoms. Additionally, a significant number of individuals in this cluster reported not experiencing sore throat or gastrointestinal symptoms such as diarrhoea, as well as loss of smell and taste, with a smaller portion affirming these symptoms were present. In the smaller cluster (Figure 7B), a completely different pattern emerges. Nearly all individuals reported not experiencing symptoms such as fever, cough, gastrointestinal issues, fatigue, and loss of smell and taste. However, almost all individuals did report dyspnoea and respiratory discomfort symptoms strongly associated with moderate to severe cases of COVID-19.

When analysing the clusters individually, both clusters show similar percentages of COVID-19 related deaths, with 18% in Cluster A and 17% in Cluster B. Recovery rates were identical, at 68%. However, in the smaller cluster, 63% of individuals were diagnosed with unspecified SARS and 36% with COVID-19, indicating that individuals without a confirmed COVID-19 diagnosis exhibited symptoms similar to those infected by SARS-CoV-2. In the largest cluster, a similar trend was observed: 44% of individuals were diagnosed with unspecified SARS, while 55% were diagnosed with COVID-19, suggesting that nearly half of the patients with unspecified SARS presented symptoms resembling those of confirmed COVID-19 cases.

In March, 45 clusters were identified. Cluster 7 contained the highest number of individuals, totalling 3,451, while Cluster 45 had the fewest, with 702 individuals. In the largest cluster, 3,293 individuals were diagnosed with COVID-19, and 158 had an unspecified SARS diagnosis. Among these, 2,425 individuals recovered, 934 died from COVID-19, for 84 the cause of death was unrecorded and 7 died for other causes. In the smallest cluster, 610 individuals were diagnosed with COVID-19, and 92 had an unspecified SARS diagnosis. Of these, 461 recovered, 208 died from COVID-19, for 26 the cause of death was unrecorded and 7 died from other causes.

Figure 8 illustrates the symptoms reported by individuals in both the largest cluster (Figure 8A) and the smallest cluster (Figure 8B) in March 2021. Analysing the data for March, a significant portion of individuals exhibited fever and cough; however, unlike during previous peak infection periods, fewer patients presented these symptoms linked to mild COVID-19 cases, as well as sore throat. Respiratory symptoms were also less frequently reported, with nearly the same percentage of individuals affected. Gastrointestinal symptoms were the least reported group overall. March was known as the most severe month for COVID-19 cases and deaths, at least within the first two years, due to specific conditions during that period. In this month, the newly identified Gamma variant spread across the country, becoming the variant most associated with infection cases and fatalities. Unlike other variants and lineages, symptoms such as fatigue, and loss of smell and taste were strongly reported among those infected, prompting more individuals, even those with mild symptoms, to seek other forms of testing, such as rapid tests, thus increasing the number of cases recorded in public health statistics. In the smallest cluster, a completely different scenario emerges. With the absence of almost all symptoms except for fever and cough symptoms widely observed among individuals infected and diagnosed with unspecified SARS (Marques et al., 2024) and the presence of dyspnoea, a symptom strongly associated with moderate to severe COVID-19 cases, it appears that a minority of individuals in this period exhibited a symptomatology indicative of significant health impairment due to the disease. This contrasts with the largest cluster, where it seems that the majority of individuals presented a range of cases, from mild to moderate and severe.

However, when analysing the clusters individually, both clusters show similar percentages of COVID-19-related deaths, with 27% in Cluster A and 28% in Cluster B. Recovery rates were also comparable, at 70% in the largest cluster and 65% in the smallest. Unlike previous periods, the majority of individuals in both clusters were diagnosed with COVID-19, accounting for 95% in the largest cluster and 86% in the smallest. Despite the higher proportion of infected individuals in both clusters compared to earlier periods, relatively fewer individuals died from SARS-CoV-2, with 27% in the largest cluster and 29% in the smallest. Regarding diagnoses of unspecified SARS, fewer patients received this diagnosis—4.5% in Cluster A and 13% in the smallest—suggesting that, due to the specific characteristics of the disease during the analysed period, the mislabelling observed in earlier periods did not occur.

In November, 17 clusters were identified. Cluster 3 contained the largest number of individuals, totalling 1,096, while Cluster 2 had the fewest, with 288 individuals. In the largest cluster, 285 individuals were diagnosed with COVID-19, and 73 had an unspecified SARS diagnosis. Among these, 858 individuals recovered, 73 died from COVID-19, and 17 died from other causes. In the smallest cluster, 53 individuals were diagnosed with COVID-19, and 235 had an unspecified SARS diagnosis. Of these, 249 recovered, 20 died from COVID-19, and 4 died from other causes.

Figure 9 illustrates the symptoms reported by individuals in both the largest cluster (Figure 9A) and the smallest cluster (Figure 9B) in November 2021. An analysis of the largest cluster reveals that only fever and cough were reported by the majority of the population; however, the presence or absence of these symptoms was not recorded. Among the remaining symptoms, only oxygen saturation and respiratory discomfort showed a notable proportion of individuals reporting them. As observed previously, gastrointestinal symptoms, along with loss of smell and taste, were not prevalent. In contrast, the smallest cluster presents a different scenario. Nearly half of the population had fever, while the other half did not. For cough, the presence of the symptom was disregarded for only a few patients. Sore throat and dyspnoea were not reported by almost any individuals, whereas respiratory discomfort was noted by nearly all patients, and the proportion of individuals with altered oxygen saturation was even higher. Gastrointestinal symptoms, as well as fatigue, loss of smell, and taste, were absent among these individuals.

When analysing the clusters individually, both clusters exhibit similar percentages of COVID-19-related deaths, with 6% in Cluster A and 7% in Cluster B. Recovery rates were also comparable, at 78% in Cluster A and 86% in Cluster B. However, unlike previous periods, a significant majority of individuals in both the largest and smallest clusters were diagnosed with unspecified SARS, at 73% and 81%, respectively. In Cluster B, where the majority of individuals did not have COVID-19, over 70% recovered from COVID-19, with both diagnoses exhibiting similar symptoms. The situation in Cluster B is particularly noteworthy, as nearly all patients presented symptoms associated with moderate to severe cases, despite not being diagnosed with COVID-19. In Cluster A, while the percentage was lower, more than half of individuals initially diagnosed with unspecified SARS also exhibited respiratory discomfort and low blood saturation factors strongly linked to respiratory complications caused by COVID-19.

## 4 Discussion

### 4.1 Limitations

A main limitation of this work is that the analysis relies solely on symptoms, which represent just one aspect of disease diagnosis, especially for COVID-19 due to its similarities with other SARS-related diseases.

While symptoms are the most visible indicators, diagnostic overlaps between SARS-CoV-2 and SARS complicate accuracy. Additionally, the dimensionality reduction technique used is sensitive to changes in hyperparameters, which is another limitation. The perplexity parameter in *t*-SNE plays a crucial role in balancing global and local structure preservation. If the perplexity is too low or too high, it can distort clusters or emphasise one structure type over the other. To address this, an optimal perplexity value should be calculated from the data, and a range of values should be tested and compared. The results presented here are based on a specific perplexity value within this range. Another limitation is the number of clusters (*k*) in k-means clustering, as the algorithm's performance depends on this choice. Results are conditioned on the selected *k* for each period, and varying *k* values can lead to different insights and interpretations based on how individuals are grouped.

In health policies, visually analysing symptoms can help identify patterns and key indicators of diseases, especially during pandemics. This approach can also reveal underreporting, as gaps in disease spread understanding affect public health. Additionally, isolation guidelines could be recommended even without lab tests by alerting patients if their symptoms closely resemble those associated with COVID-19.

### 4.2 Improvement suggestions

Based on the analysis and known similarities between COVID-19 and other SARS and viral diseases, it is not possible to rely only on symptoms to provide a diagnosis. It is essential to combine symptomatology with other information to improve the accuracy of diagnosis. Various types of patient information can be used to enhance the diagnosis.

Among the accessible information that could be added are serological tests such as IgG, IgM, and IgA, as well as antigen tests not only for COVID-19 but also for other diseases. Additionally, molecular tests such as RT-PCR could be incorporated, extending beyond COVID-19 to other diseases with similar symptoms. Besides laboratory test data, information on the patient's general health status could be included, such as pre-existing conditions, comorbidities, and vaccination history, including the number of doses and timing of administration.

In addition to the enhancement provided by the information increment, it is important to address the fact that COVID-19 and unidentified SARS cases are often confused. It remains crucial to continue examining the overlapping symptoms that cause patients in both groups to appear so similar. To advance this type of research, it is essential to eliminate the potential mislabeling issue.

A promising direction for future work would be the application of generative AI methods, i.e., Generative Adversarial Networks (GANs) or Variational Autoencoders (VAEs), which can generate more data, especially for less common diagnoses. Such methods might be able to separate those cases with unspecified SARS that have very close symptoms to COVID-19. By creating synthetic data that replicates the more subtle symptom patterns of under-represented illnesses, generative AI has the potential to provide diagnostic insight and model performance. Complications can range from overfitting to synthetic data, balancing real vs. generated data, and whether the generated data captures the subtleties of the less prevalent diseases. Additionally, ethical considerations around the utilisation of synthetic data and its impact on the explainability and trustworthiness of diagnostic models will have to be thoroughly considered.

## 5 Conclusion

The research sets out a machine learning method for visual analysis of how COVID-19 symptoms changed between various stages of the pandemics and how they were important in the characterisation of the disease during the initial two years of its outbreak in Brazil. The research sought to determine emerging trends based on comparisons with COVID-19 and other unspecified SARS patients.

From the dimensionality reduction analysis, the most important observation was the great overlap found between the two groups, wherein both subject sets created visually identical clusters in different time periods. When examined more closely, such subjects were found to be holding central positions in the clusters, which indicates that common symptoms played a significant role in the way other individuals arranged themselves around them.

Within-cluster analysis validated increased recovery and reduced mortality from COVID-19 and other disease in both cohorts. Besides symptom similarity, recovery and mortality results were strikingly similar in both cohorts. Notably, in 2020 and 2021, there was no circulation of other viruses that are the typical causes of SARS, indicating potential misclassification of cases. One troubling trend was the rising proportion of unspecified SARS. Around 50% of patients had unspecified SARS in July, and in October and November, over 70% of patients had unspecified SARS. Most had the classic COVID-19 symptoms of dyspnea, respiratory distress, hypoxemia, and cough, typical of moderate to severe COVID-19. March, which recorded the most cases and deaths, had a clear diagnostic differentiation. During off-peak seasons, however, the differentiation between COVID-19 and unspecified SARS diagnoses became progressively blurred.

This behaviour suggests that some SARS-CoV-2 infections were incorrectly classified as unspecified SARS, a suspicion corroborated not only by the symptom data but also by the other information used.

## Data Availability

The datasets presented in this study can be found in online repositories. The names of the repository/repositories and accession number(s) can be found in the article/supplementary material.
